# Healthcare Providers’ Experience Using Telehealth During the Onset of the COVID-19 Pandemic in a Predominantly Rural Patient Base: An Online Survey

**DOI:** 10.7759/cureus.36260

**Published:** 2023-03-16

**Authors:** Bashar Al Hemyari, Nicholas Coffey, Zachary W Inman, Aniruddha Singh

**Affiliations:** 1 Cardiology, University of Kentucky College of Medicine, Bowling Green, USA; 2 Bowling Green Campus, University of Kentucky College of Medicine, Bowling Green, USA; 3 Office of the Chancellor, Vanderbilt University, Nashville, USA; 4 Cardiology, Western Kentucky Heart and Lung, Bowling Green, USA

**Keywords:** cardiologists, personal satisfaction, privacy, kentucky, patient satisfaction, physicians, health care surveys, cardiology, rural population, telemedicine (tm)

## Abstract

Introduction: COVID-19 led to the rapid adoption of telemedicine with a significant spike in the literature concerning the patients’ perspective of its use. The providers’ perspective has been less well studied. Med Center Health is a healthcare network that provides services in 10 southern Kentucky counties that are home to over 300,000 people with approximately 61% of this population living in areas defined as rural. The goal of this article was to compare the experience of providers serving a predominantly rural population to their patients and compare the experience of providers between each other based on the obtained demographic data.

Methods: An online electronic survey was developed and sent out from July 13th, 2020 to July 27th, 2020 for completion to the 176 physicians of the Med Center Health Physician group. The survey gathered basic demographic information, telemedicine use during COVID-19, and perceptions of telemedicine use during and the role of telemedicine after COVID-19. Perceptions of telemedicine were gauged using Likert and Likert-style questions. Cardiology provider responses were compared to the previously published patient responses. Differences between providers were also analyzed based on the demographic data obtained.

Results: Fifty-eight providers responded to the survey with nine providers indicating that they did not use telemedicine during COVID-19. Significant differences between eight cardiologists’ and cardiology patients’ perceptions of telemedicine visits were seen for internet connectivity (p <* *0.001), privacy (p = 0.01), and clinical exam (p < 0.001) with cardiologists ranking these as more concerning or worse in all instances. These results continued when comparing perceptions of patients’ in-person experience and providers' perception of telemedicine visits with significant differences observed with clinical exam (p < 0.001), communication (p =* *0.048), and overall experience (p = 0.02). No statistically significant differences were seen between cardiologists and other providers.

Providers who indicated more than 10 years of practice rated their experience with telemedicine significantly lower in the domains of effective communication (p = 0.004), level of care provided (p = 0.02), thoroughness of clinical exam (p = 0.047), patient comfort discussing concerns (p = 0.04), and overall experience (p = 0.048). Despite this, only three providers indicated that they would not use telemedicine post-pandemic with a majority indicating that they would feel comfortable using telemedicine for follow-up visits and medication refill visits.

Conclusion: This is the first study to our knowledge to compare patient and provider satisfaction concerning telemedicine across a wide array of topics using Likert-style and Likert scale questions and the first to investigate the perception of providers who serve a predominantly rural patient base during the COVID-19 pandemic. Similar results have been found in a few previous studies concerning telemedicine being less favorably rated by more experienced providers. Further studies need to be conducted to identify and correct the barriers that exist for providers and the adoption of telemedicine.

## Introduction

In its most simplistic form, telemedicine refers to the mixture of art and science to maintain health and prevent the disease from a distance [1]. The earliest records of telemedicine arise from accounts of ancient civilizations using smoke signals to warn their neighbors of disease outbreaks. However, telemedicine has continued to evolve over time with rapid growth and development beginning in the 1960s driven by major technological advances and widespread adoption of these advances [2]. The definition of telehealth has evolved with technological advances, as Medicaid currently defines it as “two-way, real time interactive communication between the patient, and the physician or practitioner at the distant site. This electronic communication means the use of interactive telecommunications equipment that includes, at a minimum, audio and video equipment [2,3].”

COVID-19 led to a rapid rush in the development and adoption of telemedicine. Likewise, there was a corresponding bump in the literature concerning the adaptation and application of telemedicine at this time as well. A plethora of patient perspective studies were published [4]. Similarly, an increase in studies surveying providers to gain an understanding of their perspective on the rapid adaptation of telemedicine during COVID-19 happened as well. The literature concerning rapid adaptation during the COVID-19 pandemic in a predominantly rural patient base and the geographical South of the United States is scarce.

Med Center Health is a network of hospitals and medical care locations in southern Kentucky. Currently, Med Center Health has hospitals and/or medical care locations in Allen, Barren, Butler, Christian, Clinton, Hart, Logan, Monroe, Simpson, and Warren counties [5]. The Federal Office of Rural Health Policy (FORHP) is a part of the Health Resources Service and Administration of the United States Department of Health and Human Services that establishes the definition of rural that determines what areas of the United States are eligible for Rural Health funding. Based on their definition, Allen, Barren, Butler, Clinton, Hart, Logan, Monroe, and Simpson County are considered rural, while certain census tracts of Christian County are considered rural. Warren County is not considered rural [6]. In total, 346,162 individuals live in these counties based on 2010 Census data, which is what the FORHP currently bases its recommendations on [7]. Of those, approximately 61% (211,178 individuals) live in an area defined as rural by FORHP [6-8].

Besides the rural area, this distribution of practice also presented its own challenges. The counties included in Med Center Health’s scope of service had a lower percentage of households with a broadband internet subscription than either Kentucky or the United States for 2015-2019 (77.0%, 78.4%, and 82.7%, respectively). This percentage further plummets if only households in purely rural counties as defined by FORHP are considered (71.1%). Similarly, the area that Med Center Health serves has lower mean household income ($46,106, $50,589, and $62,843), a lower average percentage of people 25+ years old that are high school graduates or higher (81.5%, 86.3%, and 88.0%), the lower average percentage of people 25+ years old that have a bachelor’s degree or higher (16.3%, 24.2%, and 32.1%), and higher average percentages of individuals less than 65 years old with a disability (14.1%, 7.7%, and 10.2%) [7]. The higher level of comorbidities seen across Kentucky also complicates the implication of rapid telemedicine during COVID-19 further. Kentucky ranks fifth in the nation among states in the percentage of US adults 18+ with diagnosed diabetes [9]. This is further compounded by the second-highest rate of cigarette smoking among adults in the United States and >40 % of adults being obese [10,11].

Our study aimed to fill the current hole in the literature concerning the rapid implementation of telemedicine during the COVID-19 pandemic in a southern United States rural population. As demonstrated above, Kentucky as a whole ranks the worst in the nation in multiple indicators of health and the area served by Med Center Health presents as an especially challenging location to institute telemedicine with its low education rates, low access to broadband internet, low-income levels, and high rates of disabilities.

This article was previously presented as a meeting abstract at the 16th Annual Kentucky Chapter of the American College of Cardiology Conference & Scientific Session in September 2020.

## Materials and methods

Survey design

An online electronic survey was developed for members of the Med Center Physician Group that staffs the Med Center Health network. The Med Center Physician Group is a large multispecialty group consisting of 176 physicians. The Qualitrics© survey questionnaire was developed following the design method of Dillman et al. and was optimized for smartphone browsers [12]. The survey was preliminarily tested before being utilized. The survey was launched on July 13th, 2020 via a bulk invitation email with a direct link to the online questionnaire sent to all 176 physicians affiliated with the Med Center Physician Group. Reminder text messages and emails with a direct link to the questionnaire were sent after the first week and the day the survey closed on July 27th, 2020. Participation in the survey was voluntary and the participants were allowed to withdraw at any time. Physicians were encouraged to participate, as a donation to Med Center Health Network COVID-19 relief effort was promised for each completed survey.

The survey began with a consent form informing participants that the survey was optional and providing the aims of the survey, assurance of confidentiality, and a statement of thanks. It also indicated that their results may be excluded from the analysis if not completed. The survey then obtained basic demographic questions, including respondents’ sex, the discipline of practice, and years in practice with respondents having the options of less than 5 years, 5-10 years, or more than 10 years. Respondents were then asked if they had used telehealth during the COVID-19 pandemic to this point. The answer to this question bifurcated respondents into two different survey paths.

If the physician answered “No” regarding their participation in telehealth during the COVID-19 pandemic, the respondents were provided a list of options concerning common reasons why they did not use telemedicine and allowed a drop-down box to type their reasons if not provided in the list. The survey would then conclude, and the subject had completed the survey.

If the physician answered “Yes” regarding their participation in telemedicine during the COVID-19 pandemic, they were asked first if they had used telemedicine prior to the COVID-19 pandemic. They were then questioned concerning their preference for telemedicine modality, disadvantages to telemedicine, patient interaction satisfaction, amicability to future telemedicine use post COVID-19, the situations in which they would use telemedicine if amicable, time efficiency, and overall rating of their telemedicine experience.

As far as question-and-answer types, three different methodologies were used. The first type was unique question-and-answer types. Preference for telemedicine modality was gauged by asking first which modality providers most preferred with options of “Phone call - audio communication only,” “Face-to-face through smartphone,” and “Face-to-face using computer or tablet.” Similarly, providers were asked which modality they preferred least with the same options. They were also asked in what situations would they feel comfortable using telemedicine in the future with responses of “Follow-Up Visit,” “Medication Refills,” “Patient Preference,” “Post-Hospital Discharge Visit,” “Routine Diagnostics,” “Post-Op Appointment,” “Initial Consultation,” and “Pre-Op Appointment.”

Next, a question paired with answers of “Yes,” “No,” and “Maybe in a certain situation” with a fill-in blank for the specification of those situations was utilized. We gauged amicability to future telehealth use in this manner with the question “After restrictions due to the COVID-19 pandemic are lifted, would you consider utilizing telehealth?”.

Finally, disadvantages to telemedicine, patient interaction satisfaction, and an overall rating of their telemedicine experience were ranked using Likert-type or Likert scales. A three-point Likert-type scale was used to assess providers' concerns on issues of internet connectivity, device issues, understanding of how to use the device, understanding of how to use the platform, comfort with communicating via camera and microphone, and privacy issues. All other categories were ranked on a five-point Likert scale. The patient interaction was gauged by asking if the provider felt that they could establish a medical partnership, communicate effectively, conduct a thorough clinical exam, their perception of the patient’s comfort discussing medical concerns, their ability to relay treatment plans that the patient could understand, and provide an appropriate level of medical care.

Statistical analysis

All statistical analyses were carried out using IBM SPSS Premium, version 28.0 (IBM Corp, Armonk, NY). Differences between the distribution of answers for the same or similar Likert-type and Likert scale questions were analyzed by the Mann-Whitney U test for various patient and/or provider groups. Results were determined to be significant with a p-value less than 0.05. Patient perspective results were found and published previously by Singh et al. [13]. These results can be compared since the results of Singh et al. were found at Western Kentucky Heart and Lung (WKHL), which is a partner of the Med Center Health Network. These studies were conducted approximately at the same time point, so public perceptions and attitudes toward telemedicine and COVID-19 should be approximately the same. Finally, similar questions were asked to both groups on similar Likert and Likert-type scales, which could be directly compared in most instances.

Internal review board and participant consent

This study was conducted in conjunction with Med Center Health, the University of Kentucky College of Medicine - Bowling Green Campus, Western Kentucky Heart and Lung, and the Western Kentucky Heart and Lung Research Foundation. This study was approved by the local Institutional Review Board (20-12-06-SinA-TeleProvCOVID) prior to initiation. Participant consent was obtained by electronic consent form prior to the initiation of the survey.

## Results

Provider demographics and telemedicine use

Fifty-eight providers responded to the survey in full, corresponding to a 33% response rate. Only one provider did not complete the survey and was excluded from the analysis. Thirty-five of the 58 (60%) were male, while the rest identified as female. The most common specialties of respondents were primary care with 18 (31%) respondents and cardiology with 8 (14%) respondents. No other discipline had more than three respondents, but they included anesthesiology, dermatology, ENT, emergency medicine, family medicine, internal medicine, gastroenterology, hematology/oncology, infectious disease, neonatology, nephrology, neurology, ophthalmology, orthopedics, pediatrics, pulmonology, psychiatry, radiology, and surgery. Thirty-four (59%) providers indicated that they had been in practice for more than 10 years.

Of the 58 respondents, 9 (16%) indicated that they did not use telehealth during the COVID-19 pandemic. The most stated reason for not using telemedicine during COVID-19 was incompatibility with specialty, which was given by five respondents (56%) with unique reasons provided by the other four respondents. Among the 49 providers who indicated that they used telehealth, only 7 (14%) respondents reported using telemedicine prior to the COVID-19 pandemic. Forty-five (92%) providers identified face-to-face interactions using a computer, tablet, and/or smartphone as their most favorite telehealth service option. On the other hand, 35 (71%) providers identified phone call-audio communication-only encounters as the least preferred platform.

Cardiologists' vs patients' perspective

A Mann-Whitney U test was conducted to compare the perspectives of cardiologists and patients on telemedicine with the results presented in Table 1. There were statistically significant differences in mean for issues of internet connectivity between cardiologists (2.25) and patients (1.41), U = 168.50, z = -3.324, p < 0.001. Similarly, there were statistically significant differences in mean for issues of privacy between cardiologists (1.50) and patients (1.18), U = 280.00, z = -2.468, p = 0.01. There were also statistically significant differences in the thoroughness of the clinical exam between cardiologists (2.50) and patients (3.74), U = 699.50, z = 3.304, p < 0.001. Of note, the overall experience was also noticeably different, although not statistically significant, between cardiologists (4.25) and patients (4.51), U = 484.00, z = 0.732, p = 0.053. A proxy to determine how well each party thought the other party communicated was noticeably, but not statistically, different, when the patient question labeled PT6 (4.35) was compared to the physician question labeled CR7 (4.00) in Table 1 (U = 562.00, z = 1.716, p = 0.09). In all instances previously mentioned, cardiologists saw either the telehealth issue as more significant, such as the issue of internet connectivity and privacy, or the component of the telehealth visit as less adequate, such as thoroughness of the clinical exam, communication, and overall experience, than patients.

**Table 1:** Cardiologists' vs Patients' Telemedicine Perspective Data of cardiologists as a subgroup and the patients concerning the disadvantages of telemedicine were ranked on a three-point Likert-type scale and the perceptions of telemedicine on a five-point Likert scale with mean, standard deviation, and mean rank for each response. Cardiologists' responses are signified by the “CR” distinction and the patients' responses by the “PT” distinction. Compared responses share blocks with z-value and p-value within them.

Extent of perceived disadvantage	None/No factor (1)		Somewhat (2)			Big/Primary (3)		Median	SD	Mean rank	z-Value	p-Value
CR1 Technology issues due to poor internet connectivity	1		4			3		2.25	0.71	89.44	-3.324	<0.001
PT1 Poor internet connectivity	71		27			8		1.41	0.63	55.09		
CR2 Technology issues related to the device I was using	6		2			0		1.25	0.46	58.13	-0.076	0.94
PT2 Device technology issues	82		19			5		1.27	0.54	57.45		
CR3 My understanding of how to use the device	6		1			1		1.38	0.74	56.50	0.113	0.91
PT3 Comfort with device/software	76		21			9		1.37	0.64	57.58		
CR4 My understanding of how to use the platform	6		2			0		1.25	0.46	54.63	0.324	0.75
PT3 Comfort with device/software	76		21			9		1.37	0.64	57.72		
CR5 My comfort in communicating via camera and microphone	6		1			1		1.38	0.74	55.44	0.226	0.82
PT4 Communication issues	73		26			7		1.38	0.61	57.66		
CR6 Concern about privacy issues	4		4			0		1.50	0.54	75.70	-2.468	0.01
PT5 Privacy concerns	91		11			4		1.18	0.47	56.14		
Level of satisfaction	Extremely unsatisfied/Disagree (1)	Unsatisfied/Disagree (2)		Neutral (3)	Satisfied/Agree (4)		Extremely satisfied/Agree (5)	Mean	SD	Mean rank	z-Value	p-Value
CR7 My patient was able to communicate effectively during the telemedicine visit	0	0		2	4		2	4.00	0.756	58.80	1.716	0.09
PT6 My cardiologist seemed interested in my medical concerns	5	1		8	30		62	4.35	1.01	40.25		
CR7 My patient was able to communicate effectively during the telemedicine visit	0	0		2	4		2	4.00	0.756	58.80	1.350	0.18
PT7 My cardiologist tried to find out everything that was concerning me	7	2		5	38		54	4.23	1.09	52.31		
CR8 My patient and I were able to establish a medical partnership	0	1		0	5		2	4.00	0.926	45.88	1.134	0.73
PT8 My cardiologist was interested in establishing a medical partnership	4	2		15	45		40	4.08	0.97	58.38		
CR9 I felt that my patient understood instructions and the treatment plan at the end of the visit	0	1		0	5		2	4.00	0.926	53.81	0.352	0.41
PT9 Instructions and treatment plans were clear to me at the end of the visit	4	3		8	44		47	4.20	0.97	57.78		
CR10 I provided an appropriate level of medical care	0	0		0	5		3	4.38	0.52	58.56	-0.103	0.91
PT10 My cardiologist provided an appropriate level of medical care	5	2		8	39		51	4.23	1.01	57.42		
CR11 My patient’s clinical exam was thorough	2	3		1	1		1	2.50	1.41	23.06	3.304	0.01
PT11 My clinical exam was thorough	5	6		29	38		28	3.74	1.06	60.10		
CR12 My patient seemed comfortable discussing medical concerns	0	1		1	3		3	4.00	1.07	65.31	-0.725	0.42
PT12 I was comfortable discussing my medical concerns	4	3		7	40		52	4.25	0.98	56.91		
CR13 How would you rate the overall experience with telehealth?	0	0		0	6		2	4.25	0.46	50.00	0.732	0.053
PT13 Overall, how did you feel about your experience?	2	3		5	25		71	4.51	0.87	58.07		

Also, a Mann-Whitney U test was conducted to compare the perspectives of cardiologists on telemedicine visits and patients’ perspectives on in-person visits, which can be found in Table 2. Significant differences between cardiologists’ (4.00) and patients’ (4.45) perspectives were seen concerning communication proxy by comparing CR7 to PP1 in Table 2 (U = 527.00, z = 1.981, p = 0.048). Similarly, for perspectives on clinical exams, there were statistically significant differences between cardiologists’ (2.50) and patients’ (4.25) perspectives (U = 641.00, z = 3.369, p < 0.001). There were also statistically significant differences in perspectives on the overall experience between cardiologists’ (4.25) and patients (4.59) (U = 536.00, z = 2.250, p = 0.02). Of note, the other proxy question for communication between parties, the comparison of PP2 and CR7, was noticeably, but not statistically, different as well between cardiologists (4.00) and patients (4.42) (U = 520.00, z = 1.883, p = 0.06). In all instances previously mentioned, cardiologists rated their experience with telemedicine worse than patients.

**Table 2:** Cardiologists' Telemedicine Perspective vs Patients' In-Person Perspective Data of cardiologists as a subgroup and the patients concerning the cardiologists’ perception of telemedicine and the patients’ perception of in-person visits were ranked on a five-point Likert scale with mean, standard deviation, and mean rank for each response. Cardiologists' responses are signified by the “CR” distinction and the patients' responses are signified by the “PP” distinction. Compared responses share blocks with z-value and p-value within them.

Level of satisfaction	Extremely unsatisfied/Disagree (1)	Unsatisfied/Disagree (2)	Neutral (3)	Satisfied/Agree (4)	Extremely satisfied/Agree (5)	Mean	SD	Mean rank	z-Value	p-Value
CR7 My patient was able to communicate effectively during the telemedicine visit	0	0	2	4	2	4.00	0.756	34.63	1.981	0.048
PP6 My cardiologist seemed interested in my medical concerns	2	2	5	29	58	4.45	0.86	53.99		
CR7 My patient was able to communicate effectively during the telemedicine visit	0	0	2	4	2	4.00	0.756	35.50	1.883	0.06
PP7 My cardiologist tried to find out everything that was concerning me	2	3	7	25	59	4.42	0.91	53.92		
CR8 My patient and I were able to establish a medical partnership	0	1	0	5	2	4.00	0.926	39.19	1.446	0.15
PP8 My cardiologist was interested in establishing a medical partnership	2	1	7	34	52	4.39	0.83	53.61		
CR9 I felt that my patient understood instructions and the treatment plan at the end of the visit	0	1	0	5	2	4.00	0.926	38.06	1.580	0.11
PP9 Instructions and treatment plans were clear to me at the end of the visit	2	2	5	33	54	4.41	0.85	53.70		
CR10 I provided an appropriate level of medical care	0	0	0	5	3	4.38	0.52	46.31	0.681	0.50
PP10 My cardiologist provided an appropriate level of medical care	2	2	5	33	54	4.41	0.85	53.02		
CR11 My patient’s clinical exam was thorough	2	3	1	1	1	2.50	1.41	20.38	3.369	<0.001
PP11 My clinical exam was thorough	2	2	14	30	48	4.25	0.93	55.18		
CR12 My patient seemed comfortable discussing medical concerns	0	1	1	3	3	4.00	1.07	40.25	1.359	0.17
PP12 I was comfortable discussing my medical concerns	2	3	6	27	58	4.42	0.90	53.52		
CR13 How would you rate the overall experience with telehealth?	0	0	0	6	2	4.25	0.463	33.50	2.250	0.02
PP13 Overall, how did you feel about your experience?	1	1	6	20	68	4.59	0.75	54.08		

Cardiologists' vs other providers' experience with telemedicine

A Mann-Whitney U test was conducted to compare the perspectives of cardiologists and other providers on telemedicine, and these results are presented in Table 3. In consideration of all factors for perspectives on telemedicine, no statistically significant differences between cardiologists and other providers were observed. The most significant differences in perspective on telemedicine were the perceived level of medical care provided and overall experience. For the perceived level of medical care provided, cardiologists reported a mean score of 4.25, while other providers reported a mean score of 3.83, U = 119.00, z = -1.320, p = 0.23. For perceptions of overall experiences, cardiologists reported a mean score of 4.25, and other providers reported a mean score of 3.83, U = 121.00, z = -1.333, p = 0.26. In both instances, Med Center Health cardiologists were more satisfied than other Med Center Health providers surveyed.

**Table 3:** Cardiologists' vs Other Providers' Telemedicine Perspective Data of cardiologists as a subgroup and other providers concerning the disadvantages of telemedicine were ranked on a three-point Likert-type scale and the perception of telemedicine on a five-point Likert scale with mean, standard deviation, and mean rank for each response. Compared responses share blocks with z-value and p-value within them.

Extent of perceived disadvantage	Group	None (1)		Somewhat (2)			Big (3)		Median	SD	Mean rank	z-Value	p-Value
Technology issues due to poor internet connectivity	Cardiologist (CR)	1		4			3		2.25	0.71	25.00	-0.442	1.00
	Other providers (PR)	6		19			16		2.24	0.70	25.00		
Technology issues related to the device I was using	CR	6		2			0		1.25	0.46	26.38	-0.442	0.78
	PR	34		5			2		1.22	0.53	24.73		
My understanding of how to use the device	CR	6		1			1		1.38	0.74	27.89	-0.957	0.57
	PR	36		3			2		1.17	0.50	24.48		
My understanding of how to use the platform	CR	6		2			0		1.25	0.46	26.38	-0.442	0.78
	PR	34		5			2		1.22	0.53	24.73		
My comfort in communicating via camera and microphone	CR	6		1			1		1.38	0.74	27.06	-0.664	0.66
	PR	34		7			0		1.17	0.381	24.60		
Concern about privacy issues	CR	4		4			0		1.50	0.54	26.25	-0.311	0.80
	PR	24		15			2		1.46	0.596	24.76		
Level of satisfaction	Group	Extremely unsatisfied (1)	Unsatisfied (2)		Neutral (3)	Satisfied (4)		Extremely satisfied (5)	Mean	SD	Mean rank	z-Value	p-Value
My patient was able to communicate effectively during the telemedicine visit	CR	0	0		2	4		2	4.00	0.756	25.50	-0.120	0.93
	PR	1	3		5	23		9	3.88	0.927	24.90		
My patient and I were able to establish a medical partnership	CR	0	1		0	5		2	4.00	0.926	25.06	-0.016	1.00
	PR	0	0		6	25		10	4.10	0.625	24.99		
I felt that my patient understood instructions and the treatment plan at the end of the visit	CR	0	1		0	5		2	4.00	0.926	24.88	-0.033	0.99
	PR	0	0		4	28		9	4.12	0.577	25.02		
I provided an appropriate level of medical care	CR	0	0		0	5		3	4.38	0.52	30.63	-1.320	0.23
	PR	0	1		10	19		11	3.98	0.790	23.90		
My patient’s clinical exam was thorough	CR	2	3		1	1		1	2.50	1.41	26.69	-0.380	0.72
	PR	12	14		8	7		0	2.24	1.067	24.67		
My patient seemed comfortable discussing medical concerns	CR	0	1		1	3		3	4.00	1.07	22.94	-0.508	0.66
	PR	0	1		1	25		14	4.27	0.633	25.40		
How would you rate the overall experience with telehealth?	CR	0	0		0	6		2	4.25	0.463	30.38	-1.333	0.26
	PR	1	2		7	24		7	3.83	0.863	23.95		

Effect of years of experience on provider’s telemedicine perspective

A Mann-Whitney U test was conducted to compare the perspectives of providers with less than 10 years of experience and those with more than 10 years of experience, and these results are presented in Table 4. There were several factors with statistically significant differences between those with less than 10 years of experience compared to those with more than 10 years of experience. These factors included effective communication, level of care, thoroughness of exam, comfort in discussing concerns, and overall experience.

**Table 4:** Telemedicine Perspective of Providers With 10+ Years Experience vs 10 or Less Years Experience Data of providers who have practiced 10 years or less as a subgroup and providers who have practiced longer than 10 years concerning the disadvantages of telemedicine were ranked on a three-point Likert scale and the perception of telemedicine on a five-point Likert scale with mean, standard deviation, and mean rank for each response. Compared responses share blocks with z-value and p-value within them.

Extent of perceived disadvantage	Group	None (1)		Somewhat (2)			Big (3)		Median	SD	Mean rank	z-Value	p-Value
Technology issues due to poor internet connectivity	Providers who have practiced 10 years or less (YP)	3		10			9		2.27	0.703	24.14	0.398	0.79
	Providers who have practiced more than 10 years (OP)	4		13			10		2.22	0.698	25.64		
Technology issues related to the device I was using	YP	17		4			1		1.27	0.550	23.69	0.825	0.49
	OP	23		3			1		1.19	0.483	25.98		
My understanding of how to use the device	YP	20		1			1		1.14	0.468	26.14	-0.798	0.36
	OP	22		3			2		1.26	0.594	24.14		
My understanding of how to use the platform	YP	20		2			0		1.09	0.294	27.26	-1.425	0.12
	OP	20		5			2		1.33	0.620	23.30		
My comfort in communicating via camera and microphone	YP	17		4			1		1.27	0.550	23.57	0.902	0.45
	OP	23		4			0		1.15	0.362	26.07		
Concern about privacy issues	YP	15		6			1		1.36	0.581	27.17	-1.058	0.20
	OP	13		13			1		1.56	0.577	23.38		
Level of satisfaction	Groups	Extremely unsatisfied (1)	Unsatisfied (2)		Neutral (3)	Satisfied (4)		Extremely satisfied (5)	Mean	SD	Mean rank	z-Value	p-Value
My patient was able to communicate effectively during the telemedicine visit	YP	0	1		0	12		9	4.32	0.716	31.19	-2.803	<0.001
	OP	1	2		7	15		2	3.56	0.892	20.36		
My patient and I were able to establish a medical partnership	YP	0	0		2	13		7	4.23	0.612	27.79	-1.361	0.20
	OP	0	1		4	17		5	3.96	0.706	22.91		
I felt that my patient understood instructions and the treatment plan at the end of the visit	YP	0	0		1	14		7	4.27	0.550	27.40	-1.235	0.09
	OP	0	1		3	19		4	3.96	0.649	23.20		
I provided an appropriate level of medical care	YP	0	0		3	8		11	4.36	0.727	30.12	-2.354	0.006
	OP	0	1		7	16		3	3.78	0.698	21.16		
My patient’s clinical exam was thorough	YP	4	6		6	5		1	2.68	1.171	29.50	-1.985	0.03
	OP	10	11		3	3		0	1.96	0.980	21.63		
My patient seemed comfortable discussing medical concerns	YP	0	0		0	11		11	4.50	0.512	29.21	-2.035	0.02
	OP	0	2		2	17		6	4.00	0.784	21.84		
How would you rate the overall experience with telehealth?	YP	1	0		2	12		7	4.09	0.921	29.07	-1.979	0.048
	OP	0	2		5	18		2	3.74	0.712	21.95		

For effective communication, those with less than 10 years of experience reported a mean score of 4.286, and those with more than 10 years of experience reported a mean score of 3.607, U = 164.00, z = -2.903, p = 0.004. For the level of care, those with less than 10 years of experience reported a mean score of 4.238, and those with more than 10 years of experience reported a mean score of 4.000, U = 186.50, z = -2.354, p = 0.02. For the thoroughness of the exam, those with less than 10 years of experience reported a mean score of 2.667, and those with more than 10 years of experience reported a mean score of 2.000, U = 199.50, z = -1.985, p = 0.047. For comfort discussing medical concerns, those with less than 10 years of experience reported a mean score of 4.476, and those with more than 10 years of experience reported a mean score of 4.036, U = 205.50, z = -2.035, p = 0.04. For overall experience, there was a significant difference in those with less than 10 years of experience, 4.095, and those with more than 10 years of experience, 3.750, U = 208.50, z = -1.979, p = 0.048.

Gender’s effect on provider’s telemedicine perspective and practice

A Mann-Whitney U test was conducted to compare the perspectives of male and female providers on telemedicine, and these results are presented in Table 5. The only factor with significant differences between males and females was effective communication. For effective communication, females reported a mean score of 4.257, and males reported a mean score of 3.607, U = 162.00, z = -2.948, p = 0.003. For overall experience, there was not a significant difference between females, 4.000, and males, 3.821, U = 264.50, z = - 0.683, p = 0.50.

**Table 5:** Male Versus Female Providers' Telemedicine Perspective Data of male versus female providers concerning the disadvantages of telemedicine were ranked on a three-point Likert scale and the perception of telemedicine on a five-point Likert scale with mean, standard deviation, and mean rank for each response. Compared responses share blocks with z-value and p-value within them.

Extent of perceived disadvantage	Group	None (1)		Somewhat (2)			Big (3)		Median	SD	Mean rank	z-Value	p-Value
Technology issues due to poor internet connectivity	Self-identified male provider (MP)	6		9			13		2.25	0.799	24.46	-0.331	0.74
	Self-identified female provider (FP)	1		14			6		2.24	0.539	25.71		
Technology issues related to the device I was using	MP	23		5			0		1.18	0.390	25.30	0.255	0.80
	FP	17		2			2		1.29	0.644	24.60		
My understanding of how to use the device	MP	23		3			2		1.25	0.585	24.14	-0.798	0.43
	FP	19		1			1		1.14	0.478	26.14		
My understanding of how to use the platform	MP	21		6			1		1.29	0.535	23.46	`-1.290	0.20
	FP	19		1			1		1.14	0.478	27.05		
My comfort in communicating via camera and microphone	MP	23		4			1		1.21	0.499	25.05	0.045	0.96
	FP	17		4			0		1.19	0.402	24.93		
Concern about privacy issues	MP	17		10			1		1.43	0.573	25.89	0.581	0.56
	FP	11		9			1		1.52	0.602	23.81		
Level of satisfaction	Groups	Extremely unsatisfied (1)	Unsatisfied (2)		Neutral (3)	Satisfied (4)		Extremely satisfied (5)	Mean	SD	Mean rank	z-Value	p-Value
My patient was able to communicate effectively during the telemedicine visit	MP	1	2		6	17		2	3.61	0.875	20.29	-2.948	0.003
	FP	0	1		1	10		9	4.29	0.784	31.29		
My patient and I were able to establish a medical partnership	MP	0	1		3	17		7	4.07	0.716	25.05	0.035	0.97
	FP	0	0		3	13		5	4.10	0.625	24.93		
I felt that my patient understood instructions and the treatment plan at the end of the visit	MP	0	1		4	18		5	3.96	0.693	22.54	-1.687	0.09
	FP	0	0		0	15		6	4.29	0.463	28.29		
I provided an appropriate level of medical care	MP	0	1		5	15		7	4.00	0.770	24.41	-0.361	0.72
	FP	0	0		5	9		7	4.10	0.768	25.79		
My patient’s clinical exam was thorough	MP	8	11		6	2		1	2.18	1.056	23.82	-0.693	0.49
	FP	6	6		3	6		0	2.43	1.207	26.57		
My patient seemed comfortable discussing medical concerns	MP	0	1		1	18		8	4.18	0.670	23.79	-0.782	0.43
	FP	0	1		1	10		9	4.29	0.784	26.62		
How would you rate the overall experience with telehealth?	MP	1	1		4	18		4	3.82	0.863	23.95	-0.683	0.50
	FP	0	1		3	12		5	4.00	0.775	26.40		

Provider’s perception of the role of telemedicine after COVID

Of the 49 providers who responded that they had used telemedicine during the COVID-19 pandemic, only 3 (6%) indicated that they would not use telemedicine after the COVID-19 pandemic ended. All other providers either indicated that they would (63%) or that they would in certain situations (31%). Table 6 provides the results of predefined different scenarios that the 49 providers were likely to use telemedicine after COVID-19. A majority of providers indicated that they would feel comfortable using telemedicine for follow-up visits (88%) and medication refills (78%).

**Table 6:** Clinical Practice Circumstances After COVID in Which Providers Would Use Telemedicine Circumstances in which providers who participated in telemedicine during COVID-19 would be likely to utilize telehealth after COVID-19 are reported.

Circumstances in which you would be likely to utilize telehealth after COVID-19	No	Yes
Follow-up visit	6 (12%)	43 (88%)
Medication refills	11 (22%)	38 (78%)
Patient preference	26 (53%)	23 (47%)
Post-hospital discharge visit	29 (59%)	20 (41%)
Routine diagnostics	30 (61%)	19 (39%)
Post-op appointment	37 (76%)	12 (24%)
Initial consultation	39 (80%)	10 (20%)
Pre-op appointment	42 (86%)	7 (14%)

## Discussion

Cardiologists' and patients' experience with telemedicine

This is the first study to our knowledge to compare patient and provider satisfaction concerning telemedicine use during the COVID-19 pandemic across a wide array of subjects with Likert-style and Likert scale questions. Chang et al. did compare survey questions between providers and patients concerning telemedicine during the COVID-19 pandemic but had very limited analysis with only three Likert-scale questions [14]. Concerning our providers’ responses, they correspond to previous literature during COVID-19 in other studies conducted in the geographical Southeast of the United States. Turner et al. conducted a qualitative study in Tampa, Florida during COVID-19 that assessed mostly physicians and nurse practitioners. They found similar results in that providers found communication with patients more challenging over telemedicine. Of note, participants in this study found patient engagement lacking and that interviewing took more energy from the provider, which could be a potential cause of providers ranking patient communication significantly lower than patients' ranking of provider communication. Similarly, lack of physical examination and vital signs was noted to be a disadvantage as well [15]. Similar concerns of engagement and lack of physical examination were shared in a survey conducted by Katz et al. based in Nashville, Tennessee. They found that 72.5% of the 40 providers survey found “incomplete patient assessment” as the most concerning thing and the majority of providers were not either very satisfied or satisfied with the level of distractions in the patient’s environment during telemedicine visits. However, Katz et al. also noted that internet connectivity was the second most common top concern for surveyed providers with a 33% response rate [16]. Similar concerns about internet connectivity were noted by some Birmingham, Alabama providers in a survey conducted by Meese et al. [17].

This is the first study to our knowledge to gauge the satisfaction of providers with telemedicine during COVID-19 that serves a predominantly rural population. However, researchers at Michigan State anecdotally reported similar problems with a lack of internet access for their rural patients. They also pointed out that these patients were generally more likely to have poorer health and to be at a higher risk for adverse medical outcomes [18]. This combination could help explain the overarching negativity that providers had compared to patients concerning telemedicine in our study.

Studies conducted prior to COVID-19 to gauge satisfaction in healthcare providers who serve rural populations revealed similar results. Jordan et al. interviewed healthcare providers who managed chronic diseases in a rural native Alaskan population and found that providers liked telemedicine in this population because it allowed for more frequent patient visits and improved quality of care. On the other hand, they did find technical issues, such as lack of internet connectivity as well as lack of physical examination, to be barriers [19]. In a similar population, Ferucci et al. found that a larger percentage of providers perceived that telemedicine works “very well or well” for patients than themselves [20]. A systematic review of telemedicine in rural Native Americans found that lack of technological infrastructure was one of the largest barriers to access [21]. Another systematic review prior to COVID-19 found that privacy concerns were one of the largest barriers to physicians' adoption of telemedicine at the time [22].

Demographic effect on telemedicine perspective and practice

To our knowledge, Diaz-Miron et al. is the only other study published to show a statistically significant trend of more seasoned providers having less enjoyable experiences utilizing telemedicine during COVID-19. Specifically, they found that older surgeons in their population had similar satisfaction at the onset of COVID-19, but at a two-month follow-up, a significant downward trend in satisfaction was noted in their older surgeon base compared to younger surgeons [23]. Also, Aliberti et al. ran a noteworthy qualitative study where they looked at primary care providers' perceptions of patients 65 years or older using telemedicine during COVID-19. However, they recruited an older provider base as well with 75% of providers being in practice greater than 20 years. Most providers in this study reported that telehealth platforms should be “simplified” and that more IT support was needed. Also, Aliberti et al. noted a trend that providers who have practiced for over 20 years are more likely to indicate that they are stopping telemedicine visits after the pandemic is over [24]. Initial studies indicate that older providers face challenges in adopting telemedicine, but further research needs to be conducted to confirm this trend. Also, methods to help older providers adopt telemedicine need to be examined as well.

Limitations

The largest limitations of this study are that the providers and patients were asked varied questions and that providers from multiple disciplines were surveyed, which may inherently be a heterogeneous group instead of a homogeneous group. Concerning the varied questions limitation, our research group attempted to be as transparent as possible by providing verbatim wording of the questions provided to each group in Tables 1, 2 so readers could compare and determine their comfortability with the comparison of the results between both groups. However, we feel the questions were worded in such a manner that it is appropriate to draw comparisons between the two groups in these instances and provide a crucial look into the difference in perspective of telemedicine adoption between providers and patients. Similar studies that drew from multidisciplinary providers have been published previously and used to compare how physicians in a geographical location adopted telemedicine [25].

## Conclusions

This article helps provide insight into the experience of rural physicians in the South during the COVID-19 pandemic, which faced unique challenges compared to the rest of the United States. This quantitative inquiry via Likert-style questions found that physicians generally rated their telemedicine experience less favorably than their patient base. Also, it distinctly found that more experienced physicians rated their experience less favorably than their less experienced counterparts. However, a majority of physicians saw future use for telemedicine in clinical practice beyond COVID-19. Thus, further research needs to be done in order to increase physician experience in telemedicine as a future tool for patients and physicians alike.
